# Reducing the impact of radioactivity on quantum circuits in a deep-underground facility

**DOI:** 10.1038/s41467-021-23032-z

**Published:** 2021-05-12

**Authors:** L. Cardani, F. Valenti, N. Casali, G. Catelani, T. Charpentier, M. Clemenza, I. Colantoni, A. Cruciani, G. D’Imperio, L. Gironi, L. Grünhaupt, D. Gusenkova, F. Henriques, M. Lagoin, M. Martinez, G. Pettinari, C. Rusconi, O. Sander, C. Tomei, A. V. Ustinov, M. Weber, W. Wernsdorfer, M. Vignati, S. Pirro, I. M. Pop

**Affiliations:** 1 INFN Sezione di Roma, Roma, Italy; 2 PHI, Karlsruhe Institute of Technology, Karlsruhe, Germany; 3 IPE, Karlsruhe Institute of Technology, Eggenstein-Leopoldshafen, Germany; 4 JARA Institute for Quantum Information, Forschungszentrum Jülich, Jülich, Germany; 5 Dipartimento di Fisica, Università di Milano - Bicocca, Milano, Italy; 6 INFN Sezione di Milano - Bicocca, Milano, Italy; 7 Istituto di Nanotecnologia, Consiglio Nazionale delle Ricerche, c/o Dip. Fisica, Sapienza Università di Roma, Roma, Italy; 8 Fundación ARAID and Centro de Astropartículas y Física de Altas Energías, Universidad de Zaragoza, Zaragoza, Spain; 9 Institute for Photonics and Nanotechnologies, National Research Council, Rome, Italy; 10 INFN Laboratori Nazionali del Gran Sasso, Assergi, Italy; 11 Department of Physics and Astronomy, University of South Carolina, Columbia, USA; 12 National University of Science and Technology MISIS, Moscow, Russia; 13 Russian Quantum Center, Skolkovo, Moscow Russia; 14 IQMT, Karlsruhe Institute of Technology, Eggenstein-Leopoldshafen, Germany; 15 Institut Néel, CNRS and Université Joseph Fourier, Grenoble, France; 16 Dipartimento di Fisica, Sapienza Università di Roma, Roma, Italy

**Keywords:** Superconducting properties and materials, Superconducting devices, Single photons and quantum effects

## Abstract

As quantum coherence times of superconducting circuits have increased from nanoseconds to hundreds of microseconds, they are currently one of the leading platforms for quantum information processing. However, coherence needs to further improve by orders of magnitude to reduce the prohibitive hardware overhead of current error correction schemes. Reaching this goal hinges on reducing the density of broken Cooper pairs, so-called quasiparticles. Here, we show that environmental radioactivity is a significant source of nonequilibrium quasiparticles. Moreover, ionizing radiation introduces time-correlated quasiparticle bursts in resonators on the same chip, further complicating quantum error correction. Operating in a deep-underground lead-shielded cryostat decreases the quasiparticle burst rate by a factor thirty and reduces dissipation up to a factor four, showcasing the importance of radiation abatement in future solid-state quantum hardware.

## Abstract (web summary)

Background radiation has been identified as a key factor limiting the coherence times of superconducting circuits. Here, the authors measure the impact of environmental and cosmic radiation on a superconducting resonator with varying degrees of shielding, including an underground facility.

## Introduction

Quantum technologies based on solid-state devices are attracting a growing interest in both academic and industrial research communities, because they offer the tantalizing prospect of engineering quantum mechanical effects by using superconducting and semiconducting building blocks reminiscent of classical integrated circuits^1–3^. Although a daunting technological challenge, macroscopic components, such as capacitors, inductors, and Josephson junctions can be interconnected and assembled in complex quantum circuits, as recently proven by the operation of processors consisting of tens of quantum bits (qubits)^4–7^. While these pioneering implementations showcase the advantages of solid-state platforms, one of their main challenges for future development, increasing quantum coherence, stems from the difficulty in decoupling from various noisy environments^2^; be that dielectric defects, magnetic moments, trapped charges and vortices, spurious electromagnetic modes, or excess quasiparticles (QPs).

QPs, which can be viewed as broken Cooper pairs, degrade the performance of superconducting circuits in two ways^8^: their presence introduces dissipation, and fluctuations in their numbers give rise to noise. Although QPs are particularly damaging in circuits employing the high kinetic inductance of Cooper pairs^9–11^, often constituting the dominant source of decoherence, we will argue below that QPs can be an indicator of a more generally damaging pair-breaking mechanism for solid-state hardware, namely radioactivity.

**Fig. 1:**
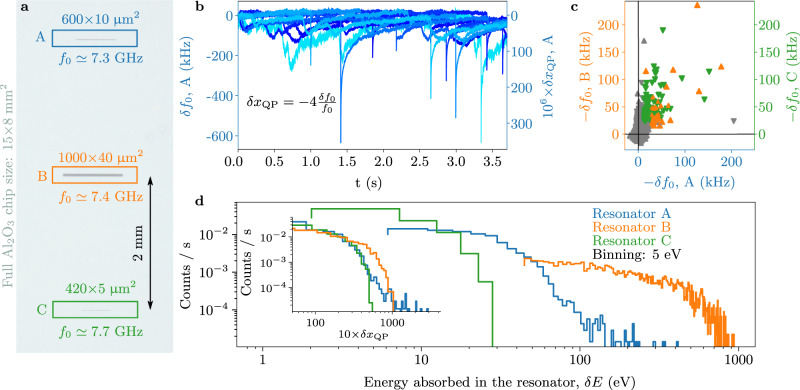
Quasiparticle bursts and deposited energy in grAl resonators. **a** False-colored photograph of the central part of the sapphire chip, supporting three 20 nm thick grAl resonators, labeled A, B, and C. **b** Overlay of ten measured time traces for the resonant frequency shift *δf*_0_ of resonator A. Similarly to refs. ^10,21,36^, quasiparticle (QP) bursts appear as sudden drops, given by the sharp rise in kinetic inductance, followed by a relaxation tail. The *y*-axis on the right-hand side shows the corresponding fractional quasiparticle density shift *δx*_QP_ = −4*δf*_0_/*f*_0_. We identify a QP burst by applying a derivative filter, triggering only on sharp rises in the baseline. For clarity, the shown traces are selected to contain a QP burst; on average, only one trace in ten contains a QP burst. To highlight the fact that QP bursts are correlated in time, in **c**, we plot the measured frequency shifts of resonator B (upward triangles) and C (downward triangles) versus the frequency shift of resonator A. Colored markers correspond to values above threshold, with the threshold defined as two standard deviations of the baseline fluctuations (cf. Supplementary Information). Therefore, each colored marker depicts a time-correlated QP burst between resonators A–B (orange) and A–C (green). **d** Estimated distribution of the energy absorbed in the resonators *δE* = *δx*_QP_Δ_grAl_*n*_CP_*V*, calculated from the measured *δx*_QP_ shown in the inset, where Δ_grAl_ ≃ 300 μeV is the grAl superconducting gap, and *n*_CP_ = 4 × 10^6^ μm^−3^ is the volume density of Cooper pairs, and *V* is the volume of each resonator. For each burst, the energy deposited in the substrate is estimated to be 10^3^–10^4^ times greater than *δE* (cf. Supplementary Information). The total QP burst rate Γ_*B*_ is obtained by counting all bursts above the common threshold *δx*_QP_ = 5 × 10^−5^.

High-energy particles are a routinely observed source of noise in low-temperature circuits, such as microcalorimeters^12,13^, bolometers^14^, and MKIDs^15^. In particular, the latter ref. reports time-correlated glitches in the resonant frequency of an array of several hundreds same-chip resonators. Moreover, ref. ^16^ has also provided a clear evidence that radioactivity induces errors correlated both in space and time in qubits, undermining many algorithms for quantum error correction^17^, and ref. ^18^ has recently shown that the coherence limit imposed by ionizing radiation for transmon type qubits is in the millisecond range, only one order of magnitude above the state-of-the-art. As dielectric losses are steadily decreased^19,20^, further improving the coherence of solid-state devices will soon hinge on the reduction of QPs, and more generally on ionizing radiation abatement. In thermal equilibrium, at typical operational temperatures of 20–50 mK, QPs should be an extremely rare occurrence in commonly used materials such as Al and Nb, with critical temperatures well >1 K: e.g., one would need to wait a time comparable to the age of the universe to observe a single thermal QP in a 10 μm^3^ volume of Al at 100 mK. However, the detrimental effects of nonequilibrium QPs are routinely observed in a variety of devices^9,21–32^, including the microwave resonators used in this work (cf. Fig. 1). The multifarious QP sources include stray infrared radiation^24,32^, high-power microwave drive^33^, and pair-breaking phonons in the device substrate^34,35^, resulting from environmental or cosmic radioactivity. The latter is potentially damaging for any solid-state quantum hardware, not only superconducting, as it can give rise to correlated energy bursts in devices on the same chip. In the case of superconducting resonators, for instance, phonons generated by particle impacts in the device substrate produce correlated QP spikes orders of magnitude above the baseline^36,37^, visible as abrupt frequency drops (see Fig. 1b, c). The correlation among events can be mitigated by using so-called phonon traps^15,35^. On the contrary, their absolute rate (today of the order of one every few seconds^9,10,36^) can only be reduced by a careful selection of radio-pure materials and shielding protocols. Without a mitigation strategy, the ensuing relatively long-lasting^38^ and correlated^36,37^ effects of radioactive interactions can hinder quantum error correction protocols.

Superconducting circuits can be sensitive to a variety of radioactive sources, depending, among others, on the distance from the device, penetrating power, spectral distribution, and shielding. So-called far sources consist of cosmic rays, mainly sea-level muons at a rate of ~1 cm^−2^ min^−1^ (refs. ^39,40^), as well as decay products of location-specific contaminants. Even when far sources can be shielded, using, e.g., Pb screens or underground facilities, near sources, such as residues from handling and machining, or radioactive isotopes in the sample holder and the sample itself might need to be mitigated by material selection and decontamination. In this work, we demonstrate that by reducing radioactivity we lower the internal dissipation in superconducting microwave resonators by factors two to four, and the QP burst rate by a factor 30. This was achieved by a combination of material selection and cleaning, and by shielding under the 1.4 km granite layer at the Gran Sasso National Laboratory (L’Aquila, Italy), corresponding to a 3.6 km water equivalent.

## Results and discussion

We use high kinetic inductance granular aluminum (grAl) superconducting resonators (see Fig. 1a) as a sensitive QP probe, following the principle of kinetic inductance detectors (KIDs)^41^. Shifts in their resonant frequency ${f}_{0}^{-1}=2\pi \sqrt{LC}$, where *C* is the capacitance of the mode, directly reflect changes in the inductance *δL*/*L* = −(2/*α*)*δf*_0_/*f*_0_, where *α* is the ratio of kinetic inductance over the total inductance. In the case of high kinetic inductance materials, such as grAl, where the geometric inductance can be neglected^10^, the measured relative frequency shift informs on the corresponding change in the number of QPs with respect to the number of Cooper pairs: *δx*_QP_ = 2*δL*/*L* = −4*δf*_0_/*f*_0_. Both KIDs and qubits are sensitive to pair-breaking phonons produced by radioactive deposits in the chip. The major difference between these devices is the phonon absorption probability in a region of the device susceptible to QPs, which depends on the supercurrent mode volume. We decided to rely on KIDs because, apart from the ease in operation, they enable a real-time monitoring of the QPs bursts due to phonon absorption, and they are sensitive over a much wider energy range compared to qubits.

The resonators were fabricated using optical lithography on a 1.2 cm^2^ and 330 μm thick sapphire substrate, following the stripline design of ref. ^10^. Their dimensions and corresponding resonant frequencies *f*_0_ are listed in Fig. 1a. We performed quality factor measurements in the range of $\bar{n}=1$ circulating photons, which are the typical conditions for quantum circuits, and time domain evolution measurements of the resonant frequency at the highest available power before bifurcation. Furthermore, we used a 3D waveguide sample holder^42^ in order to minimize the electric field density at the interfaces and reduce coupling to dielectric losses^43^. In this setup, losses in grAl resonators are dominated by nonequilibrium QPs and dielectric losses contribute to up to ~20% (refs. ^10,35^). In Fig. 1b, we show typical time traces for the frequency shift *δf*_0_ of resonator A, measured in a cryostat above ground. We observe abrupt drops of *f*_0_, indicative of a QP burst in the resonator film, followed by a relaxation tail, associated with QP recombination and diffusion, similarly to refs. ^10,21,36,37^, one every ~10 s. We interpret them as the aftermath of ionizing events in the substrate, causing an energy release in the form of pair-breaking phonons, which in turn produce QPs. Indeed, as shown in Fig. 1c and in Supplementary Information, most QP bursts in resonator A are correlated with those in resonators B and C, proving the key role played by substrate phonons^37^. Notice that although resonator C is twice as far from resonator A compared to B, the correlation plot does not appear qualitatively different, indicating that in our present geometry QP bursts are time-correlated over at least 10 mm^2^ areas of the chip, similarly to refs. ^36,37^. The histogram of the QP burst rate as a function of the energy absorbed in the resonators is shown in Fig. 1d. We estimate the efficiency of phonon absorption from the substrate into the resonators to be 10^−3^–10^−4^, placing the energy deposited in the substrate by each ionizing impact in the keV–MeV range (cf. Supplementary Information).

**Fig. 2:**
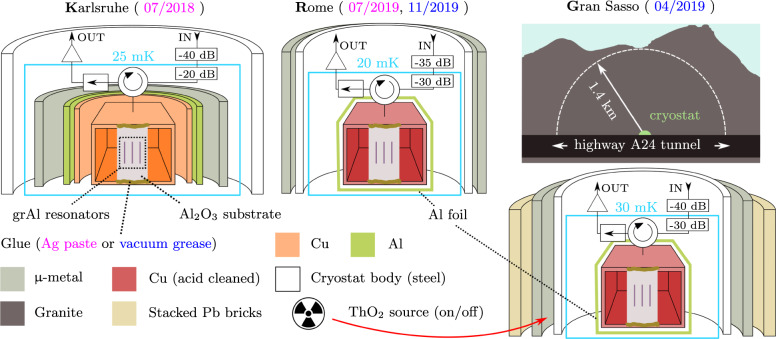
Three different setups with various degrees of shielding against ionizing radiation. Schematic half-sections of the setups, in Karlsruhe, Rome, and Gran Sasso, denoted *K*, *R*, and *G*, respectively. The measurement dates for each setup are indicated in the top labels. The sapphire chip is glued to a copper waveguide using either silver paste (K and R, magenta) or vacuum grease (G and R, blue). A circulator routes the attenuated input signal to the sample holder, and the reflected output signal to an isolator and an amplification chain (cf. Supplementary Information). In the R and G setups, the waveguide is etched with citric acid to remove possibly radioactive contaminants. The G setup, located under 1.4 km of granite (3.6 km water equivalent) is operated in three configurations. First, the cryostat is surrounded by a 10 cm thick wall of lead bricks. Two days later, the bricks were removed. Finally, we added a ThO_2_ radioactive source next to the cryostat body (cf. red arrow).

**Fig. 3:**
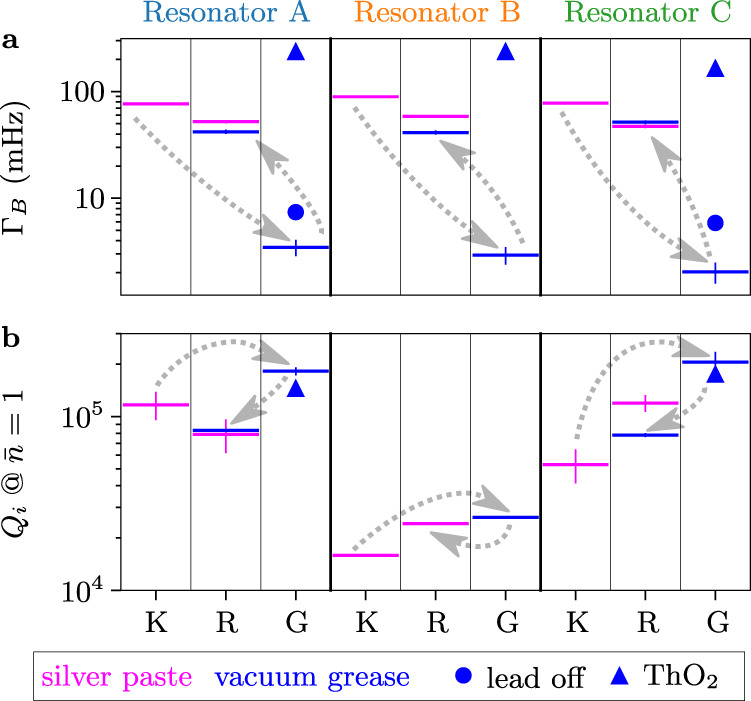
Effect of ionizing radiation shielding on resonator performance. **a** Quasiparticle burst rate Γ_*B*_ and **b** internal quality factor at single photon drive *Q*_*i*_ for all resonators and setups. When the sample is cleaned and tested in the R setup, the measured Γ_*B*_ and *Q*_*i*_ values are comparable to those obtained in K. Measurements in the G setup show a reduction in QP burst rate Γ_*B*_ (factor 30) and dissipation (up to a factor 4). In G, removing the lead shielding increases Γ_*B*_ by a factor two. Adding a ThO_2_ radioactive source next to the cryostat body yields a Γ_*B*_ greater than the one measured above ground, and decreases the internal quality factor *Q*_*i*_ by 18 ± 3%. Error bars are $\sqrt{N}$ for Γ_*B*_ (Poissonian error) and standard deviations of all data points in the $\bar{n}=0.1{-}10$ range for *Q*_*i*_ when available, and are not shown when smaller than the marker size. The chronological order of measurements in the three different setups is indicated by the dotted gray arrows.

In the following, we will use the QP burst rate as an indicator of the ionizing radiation flux, while we perform various combinations of material selection, cleaning, and shielding. The three setups, located in *K*arlsruhe, *R*ome, and *G*ran Sasso, denoted by K, R, and G, are schematized in Fig. 2, and the dates of the four measurement runs are indicated by the top labels. The corresponding measured QP burst rates and internal quality factors of the resonators are listed in Fig. 3.

Both the K and R setups are located above ground. The K setup is typical for superconducting circuit experiments and features additional magnetic shielding compared to the G and R setup, consisting of a superconducting and a μ-metal barrel encasing the waveguide. In the K and R facilities we expect muons (and their secondary products) to hit the substrate with a rate of ~0.6 mHz. These interactions release an average energy of 0.8 MeV, with tails extending up to few MeV. In the G setup, the 1.4 km of rock overburden reduces the cosmic ray flux by six orders of magnitude and thus to a completely negligible rate in the chip. However, according to our simulations (cf. Supplementary Information), an important contribution to the measured rate comes from radioactive contamination in the laboratory environment. According to measurements performed with a NaI commercial spectrometer, we predict a rate of 16 mHz in K, 48 mHz in R, and 4 mHz in G. The average energy deposit due to environmental radioactivity is 0.1 MeV.

The burst rate and the internal quality factors measured for all resonators in the three sites are shown in Fig. 3. The first set of data was collected in K, where we measured a burst rate (averaged over the three resonators) of ~76 mHz. In the K setup, no specific radio-purification measures have been taken. Therefore, the factor four larger burst rate compared to the expected value from the NaI spectrometer is likely due to residual radioactive contamination of the sample holder and its immediate environment. For the next data sets, we cleaned the sample enclosure and its mounting parts with citric acid and hydrogen peroxide to reduce surface contamination, removed the potentially radioactive indium wire used to seal the copper cap, substituted lead soldering with araldite glue, and replaced silver paste with more radio-pure^44^ cryogenic grease to attach the chip to the copper holder. The sample placed in this radio-cleaned holder was measured deep underground in a cryostat surrounded by a wall of 10 cm thick Pb bricks. The rate induced by muons in the underground setup is very small compared to the one induced by environmental radioactivity; however, the effectiveness of lead shielding against the environmental radioactivity increases when going underground. Cosmic rays, indeed, produce gamma showers in the materials surrounding the sample (including the lead shield itself) that are hard to suppress above ground. Thus, the same lead shield offers a stronger reduction against gamma’s when used deep underground. According to our simulations, the lead shield suppresses the contribution of environmental radioactivity to the counting rate down to 0.5 mHz.

We measured an average QP burst rate of 2.6 mHz, pointing to a small residual contribution due to contamination of the dilution refrigerator, and of the materials of the sample holder and assembly. The cleaning protocols and the cryostat shielding could be largely improved; nevertheless, we observed a reduction in the burst rate by a factor 30.

We performed two additional measurements by first removing the bricks, and then exposing the cryostat to a ^232^Th source in the form of ThO_2_. Removing the lead shielding increases the burst rate by a factor two, and adding the ThO_2_ source increases the rate beyond above ground levels, confirming the radioactive origin of the bursts. The internal quality factors (cf. Fig. 3, bottom) are anticorrelated with the burst rates between above and underground measurements, achieving up to a fourfold increase in the G setup. The measurement of single photon quality factors consists of averaging multiple frequency domain traces over a time span of tens of minutes, during which the resonator is averaging over QP-induced losses.

To confirm that such improvement in the internal quality factor was not due to the chip ageing, or to the electronics or magnetic shielding used in the G site, we moved the “cleaned” assembly with the entire readout and magnetic shield to the above ground cryostat in R. As expected, we observed an increase in the burst rate and a corresponding worsening in the quality factor. We also investigated if using vacuum grease instead of silver paste to attach the chip to the copper holder could affect the thermalization of the substrate and thus the quality factor. We replaced the vacuum grease with silver paste and repeated the measurements obtaining consistent results; a small excess in the burst rate was observed, probably related to contamination in the silver paste.

Notice that the burst rate is not simply a proxy for the quality factor, as indicated by the fact that the quality factor in the G setup only decreased by ~20% when the QP burst rate was increased by two orders of magnitude by using the ThO_2_ source. This is not surprising, given the fact that the ThO_2_ source alone is not a good proxy for the radioactive contributions that we would expect above ground, where we have to account for muons, releasing an average energy seven to eight times larger than the average energy released by the ThO_2_ source, and for neutrons. Therefore, a survey of various sources is needed in order to quantitatively understand the QP generation, and the influence on the quality factors of superconducting devices. We would like to mention that, during the editing stages of our work, in ref. ^45^ the authors report QP bursts with orders of magnitude higher rate, which decays over time scales of several weeks, and of undetermined origin, showcasing the challenges that lie ahead for the community.

In conclusion, we showed that the performance of superconducting circuits at the current level of coherence can be significantly degraded by environmental radioactivity in a typical above ground setting, in particular due to ionizing interactions in the device substrate. We demonstrated that the rate of correlated QP bursts is reduced by up to a factor 30 by shielding in a deep-underground facility and by a radioactive decontamination in the near environment of the sample. Furthermore, the quality factors of high kinetic inductance superconducting resonators improved up to a factor four with respect to above ground values.

These observations highlight the need for a systematic assessment of radioactive sources, which can produce energy bursts in solid-state quantum hardware, as well as for a better understanding of the relevant chains of mechanisms, such as the creation of electron–hole pairs, and the excitation of high-energy phonons, which potentially limit the performance of superconducting and semiconducting devices. The effectiveness of radiation abatement and phonon damping solutions, such as phonon traps^15,35^, will determine whether the next generation of solid-state quantum processors will need to be operated in deep-underground facilities.

## Supplementary information

Supplementary Information

## Data Availability

All relevant data are available from the authors upon request.
