# Supplementary material for: Reducing the impact of radioactivity on quantum circuits in a deep-underground facility
Source: Nat Commun. 2021 May 12;12:2733. doi: 10.1038/s41467-021-23032-z (PMC8115287; doi:10.1038/s41467-021-23032-z)
Supplement: Supplementary file 1 — Supplementary Information [file 41467_2021_23032_MOESM1_ESM.pdf]

Supplementary Information:
Reducing the impact of radioactivity on quantum circuits in a deep-underground facility

L. Cardani,[1,*] F. Valenti,[2,3,†] N. Casali,[1] G. Catelani,[4] T. Charpentier,[2] M. Clemenza,[5,6] I. Colantoni,[1,7] A. Cruciani,[1] G. D'Imperio,[1] L. Gironi,[5,6] L. Grünhaupt,[2] D. Gusenkova,[2] F. Henriques,[2] M. Lagoin,[2] M. Martinez,[8] G. Pettinari,[9] C. Rusconi,[10,11] O. Sander,[3] C. Tomei,[1] A. V. Ustinov,[2,12,13] M. Weber,[3] W. Wernsdorfer,[2,14,15] M. Vignati,[1,16] S. Pirro,[10] and I. M. Pop[2,14,‡]

[1]*INFN Sezione di Roma, 00185 Roma, Italy*
[2]*PHI, Karlsruhe Institute of Technology, 76131 Karlsruhe, Germany*
[3]*IPE, Karlsruhe Institute of Technology, 76344 Eggenstein-Leopoldshafen, Germany*
[4]*JARA Institute for Quantum Information, Forschungszentrum Jülich, 52425 Jülich, Germany*
[5]*Dipartimento di Fisica, Università di Milano - Bicocca, 20126 Milano, Italy*
[6]*INFN Sezione di Milano - Bicocca, 20126 Milano, Italy*
[7]*Istituto di Nanotecnologia, Consiglio Nazionale delle Ricerche, c/o Dip. Fisica, Sapienza Università di Roma, 00185, Roma, Italy*
[8]*Fundación ARAID and Centro de Astropartículas y Física de Altas Energías, Universidad de Zaragoza, Zaragoza 50009, Spain*
[9]*Institute for Photonics and Nanotechnologies, National Research Council, 00156 Rome, Italy*
[10]*INFN Laboratori Nazionali del Gran Sasso, 67100 Assergi, Italy*
[11]*Department of Physics and Astronomy, University of South Carolina, 29208 Columbia, USA*
[12]*National University of Science and Technology MISIS, 119049 Moscow, Russia*
[13]*Russian Quantum Center, Skolkovo, 143025 Moscow, Russia*
[14]*IQMT, Karlsruhe Institute of Technology, 76344 Eggenstein-Leopoldshafen, Germany*
[15]*Institut Néel, CNRS and Université Joseph Fourier, Grenoble, France*
[16]*Dipartimento di Fisica, Sapienza Università di Roma, 00185, Roma, Italy*
(Dated: April 6, 2021)

In this Supplementary Information we provide detailed information on the microwave schematics of the three setups (K, R, and G), the sample mounting and shielding, the frequency drift of the resonators in time, the measurement of correlated quasiparticle (QP) bursts, the method employed to choose the bin size for the histograms of the energy absorbed by the resonators, the size distribution of QP bursts, the additional calibration experiments and simulations used to estimate the efficiency of phonon absorption from the substrate to the resonators, and the evaluation of the effect of background sources.

* laura.cardani@roma1.infn.it
† First two authors contributed equally.
‡ ioan.pop@kit.edu

Supplementary Note 1: Schematics of microwave setups

We show schematics of the microwave wiring for the K, R, and G setups in Supplementary Figure 1. The three setups are similar, with the notable difference that K is equipped with microwave and IR filters.

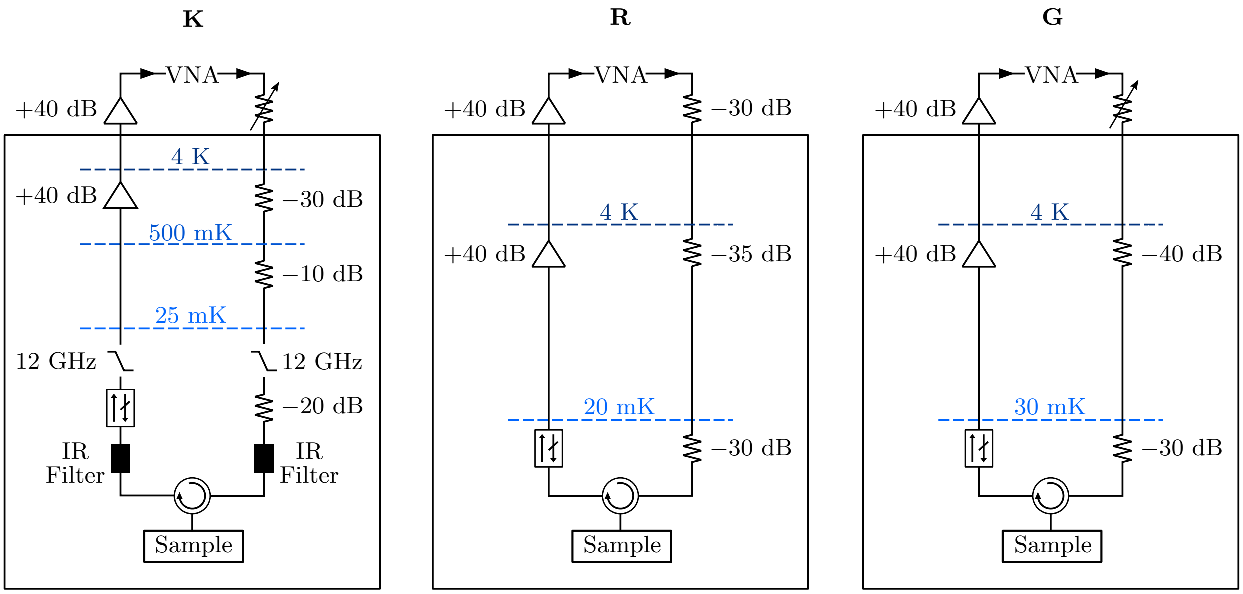

Supplementary Figure 1. **Schematics of the input and output lines of the K, R and G setups**. The displayed components are thermalized to the nearest temperature stage indicated above them.

Supplementary Note 2: Sample mounting and cryostat shielding

We show the sample mounting and the dilution cryostat of the G setup in Supplementary Figure 2.

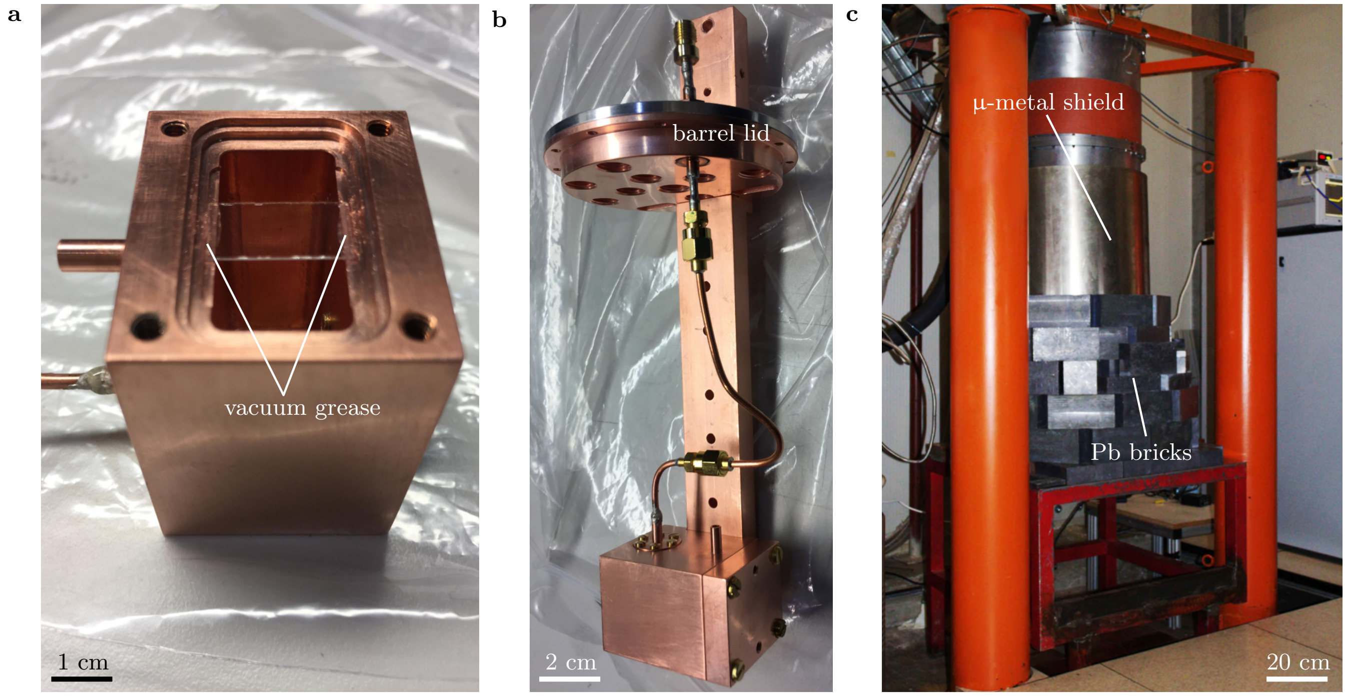

Supplementary Figure 2. **Sample mounting and cryostat shielding**. **a**, Copper waveguide without the cap. The sapphire chip is glued to the waveguide with vacuum grease (shown) or Ag paste. **b**, Mounting copper rod with capped waveguide screwed on. In the K setup, a Cu/Al bilayer barrel and a μ-metal barrel are screwed onto the lid, encapsulating the rod-waveguide ensemble. **c**, Dilution cryostat in the G setup. Notice the ∼2 mm thick μ-metal barrel and the wall of $\sim 20 \times 10 \times 5$ cm^3 Pb bricks surrounding it.

Supplementary Note 3: Frequency drift of the resonators over time

We report the frequency drift over time of the three grAl resonators in Supplementary Figure 3. During a cooldown, the resonator frequency does not change; between cooldowns the chip is stored at room temperature and atmospheric pressure. The reduction in resonant frequency is similar to the commonly observed ageing of Josephson junctions, and it is related to changes in the oxidation of the film over time, which are strongly influenced by organic contaminants [1, 2].

**Setup**	**Resonant frequency (GHz)**		
	Resonator A	Resonator B	Resonator C
K	7.293158	7.465360	7.709244
G	7.243984	7.419364	7.658760
R, vg	7.239192	7.415607	7.654537
R, sp	7.193234	7.369042	7.610113

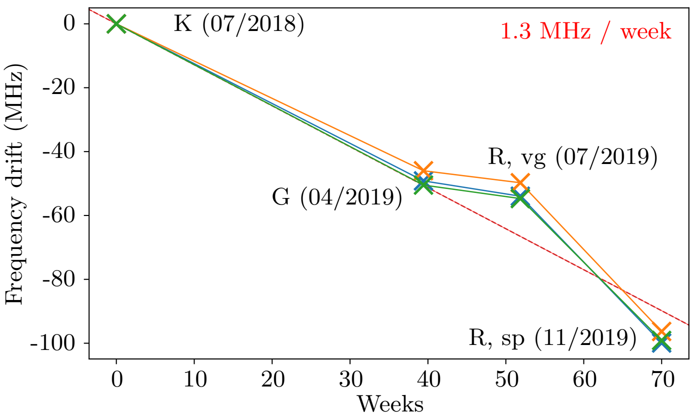

Supplementary Figure 3. **Frequency drift**. Time evolution of the resonant frequencies of resonators A, B, and C over the four measurements runs. As a reference, we plot a linear negative drift of 1.3 MHz per week (red dashed line).

Supplementary Note 4: Measurement of correlated bursts with a VNA

In order to investigate whether QP bursts are correlated in time, we measure time multiplexed traces of the phase response of two resonators by employing a Keysight E5071C Vector Network Analyzer (VNA). The method is summarized in Supplementary Figure 4. The time needed to switch between the two frequencies is the inverse of the IF bandwidth, 1/100 Hz = 10 ms. The time interval Δt between each pair of measured points is dominated by the time needed to transfer their values from the VNA to the measurement PC. We estimate it by dividing the total acquisition time by the number of acquired 2-point measurements, giving an effective sampling period $\Delta t \approx 0.3$ s.

As discussed in the main text, the relaxation time after a QP burst is from tens to hundreds of milliseconds, depending on the setup. This time is short compared to the sampling period Δt. This method, while it has the advantage of being conceptually simple and possible to implement with off-the-shelf electronics, is restricted to measuring only two resonators at a time, and is clearly limited when it comes to evaluating the size of QP bursts and their exact position in time. These limitations can be overcome by using frequency domain multiplexing and custom designed electronics, similarly to Refs. [3–6].

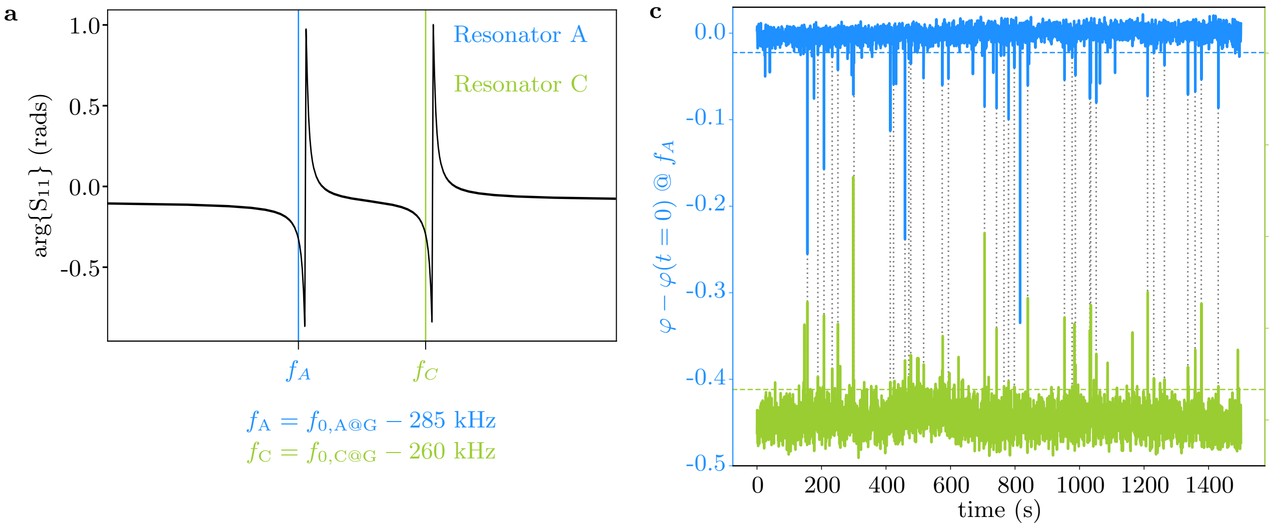

Supplementary Figure 4. **Time multiplexed measurement of the phase response of two resonators using a Vector Network Analyzer (VNA).** **a**, The phase response of the reflected signal vs. frequency (black line) is sampled at two points in the vicinity of the resonant frequency of the two resonators (blue and green). **b**, Time multiplexed phase response $\varphi_{A,C}(t) = \arg\{S_{11}(f_{A,C}, t)\}$ for resonators A and C vs. time in G with the ThO_2 source. For clarity, we invert the sign of the right-hand y axis. The time interval between measurements at the two different frequencies is 10 ms. Bursts exceeding the two standard deviation threshold (horizontal dashed lines) for both resonators are highlighted by vertical dotted gray lines. The relaxation tail after the QP bursts is not resolved due to the fact that the time interval between successive measurements in each trace is relatively long, $\Delta t \approx 0.3$ s (see text for details).

Supplementary Note 5: Measurement of the internal quality factor

We fit the complex reflection coefficient S_{11} of the resonators with the procedure detailed in Ref. [7] in order to extract the internal and coupling quality factors and the resonant frequency. We compute the average number of drive photons circulating in the resonators as $\bar{n} = 4P_{\mathrm{cold}}Q_l^2/(\hbar\omega_0^2 Q_c)$, where P_{cold} is the VNA probe power minus the nominal attenuation on the line down, Q_l and Q_c are the loaded and coupling quality factors, and ω_0 is the resonant frequency in radians per second. We set the IF bandwidth of the VNA to 10 kHz and we average for 500 times. We plot the results in Supplementary Figure 5.

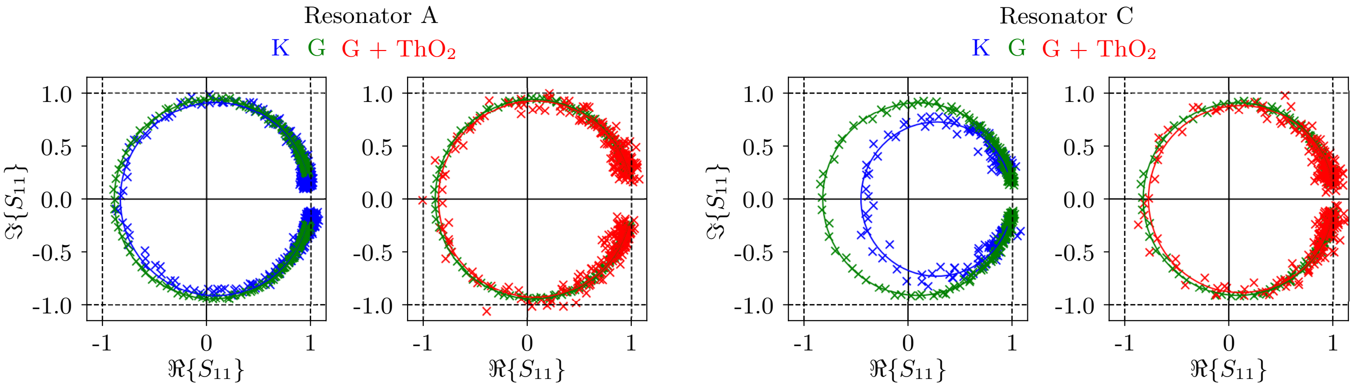

Supplementary Figure 5. **Measurement of the internal quality factor.** Reflection coefficient of resonator A (left) and C (right), normalized to the sample holder response and plotted in the complex plane. We show data for both resonators in G (green) compared to those in K (blue) and in G with the Pb shield removed and ThO_2 source added (red). Crosses and solid lines indicate the raw data and the circle fit from Ref. [7], respectively.

Supplementary Note 6: Choosing the bin size for the QP bursts energy distribution

In Fig. 1d in the main text, we show a distribution of QP bursts vs. the energy absorbed by the resonators. In the following we describe the method used to define a common bin size ΔE for the distribution of QP bursts. The goal is to find the smallest ΔE that is larger than the uncertainty corresponding to the phase response uncertainty $\Delta\varphi$ for all resonators. The phase response of the reflected signal vs. frequency obeys

$$\varphi(f) = \arctan\left(2Q_l \frac{f_0 - f}{f_0}\right), \tag{1}$$

where f_0 is the resonant frequency, and Q_l is the loaded quality factor (cf. Supplementary Figure 6a). The response around $f_0 + \delta f_0$ is linear for $\delta f_0 \to 0$ and asymptotic to $\pm\pi$ for $\delta f_0 \to \mp\infty$: the greater the shift δf_0 corresponding to large QP bursts, the greater the frequency uncertainty corresponding to $\Delta\varphi$. Thus, we can only quantitatively trust shifts δf_0 up to the point where the phase uncertainty $\Delta\varphi$ corresponds to a frequency difference smaller than the bin size in frequency units, Δf_0. This places the following constraints:

$$\begin{aligned} &|\varphi(f_0 + \delta f_0) - \varphi(f_0 + \Delta f_0 + \delta f_0)| < \Delta\varphi \\ \iff &\left|\varphi\left(f_0 + \delta E \frac{f_0}{4V\Delta_0 n_{\mathrm{CP}}}\right) - \varphi\left(f_0 + \Delta E \frac{f_0}{4V\Delta_0 n_{\mathrm{CP}}} + \delta E \frac{f_0}{4V\Delta_0 n_{\mathrm{CP}}}\right)\right| < \Delta\varphi, \end{aligned} \tag{2}$$

where Δf_0 and ΔE are the bin sizes in frequency and energy units respectively, $\delta E = \delta x_{\mathrm{QP}} n_{\mathrm{CP}} \Delta_0 V$ is the burst in energy units and $\delta x_{\mathrm{QP}} = -4\delta f_0/f_0$ is the burst in fractional QP density units, V is the volume of the resonator, $\Delta_0 \simeq 300$ µeV is the superconducting gap of grAl, $n_{\mathrm{CP}} = 4 \times 10^6$ µm^{-3} is the Cooper pair density of Al, and f_0 is the unperturbed resonant frequency.

In order to calculate the uncertainty of the response $\Delta\varphi$, we substract the fitted arctangent dependence of Supplementary Eq. (1) from the measured $\arg\{S_{11}\}$ (cf. Supplementary Figure 6a), and we compute its nearest point difference φ'. We define $\Delta\varphi = 2\ \mathrm{stdev}(\varphi')$ (cf. Supplementary Figure 6b), and we find $\Delta\varphi \lesssim 10$ milliradians for all resonators.

Finally, using $\Delta E = 5$ eV, Supplementary Eq. 2 is satisfied for $\Delta\varphi = 10$ milliradians and for all measured QP burst in all resonators.

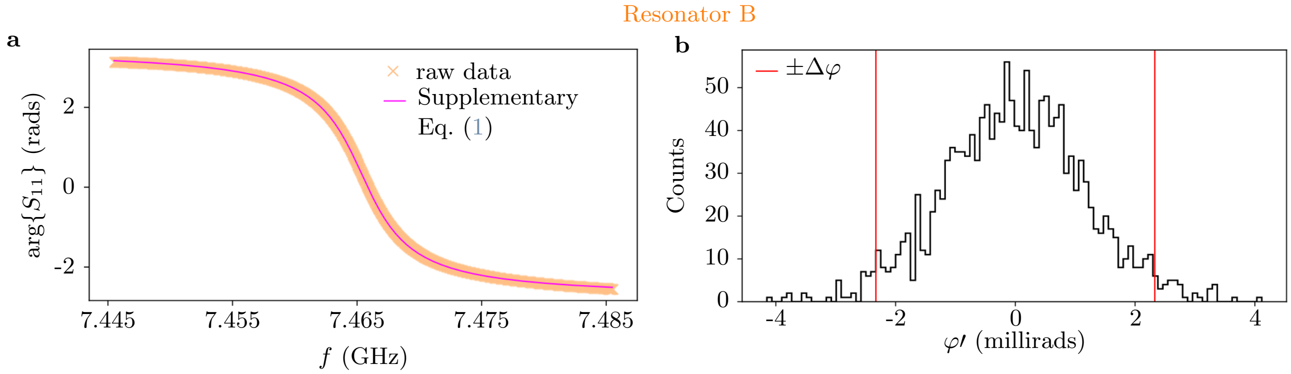

Supplementary Figure 6. **Phase response vs. frequency and phase uncertainty for resonator B**. **a**, Measured phase response vs. frequency (orange crosses) fitted with Supplementary Eq. (1) (magenta line). **b**, Distribution of the nearest point differences φ' of the measured phase response with the arctangent component removed.

Supplementary Note 7: Distribution of QP bursts in R and G setups

We report the distribution of QP bursts for resonators in R and G, to complement the distribution for resonators in K shown in the main text (Fig. 1d, inset). We omit the distribution of bursts in G with the Pb shield present, resulting in the lowest burst rate (one every 10 minutes) due to the lack of sufficient statistics.

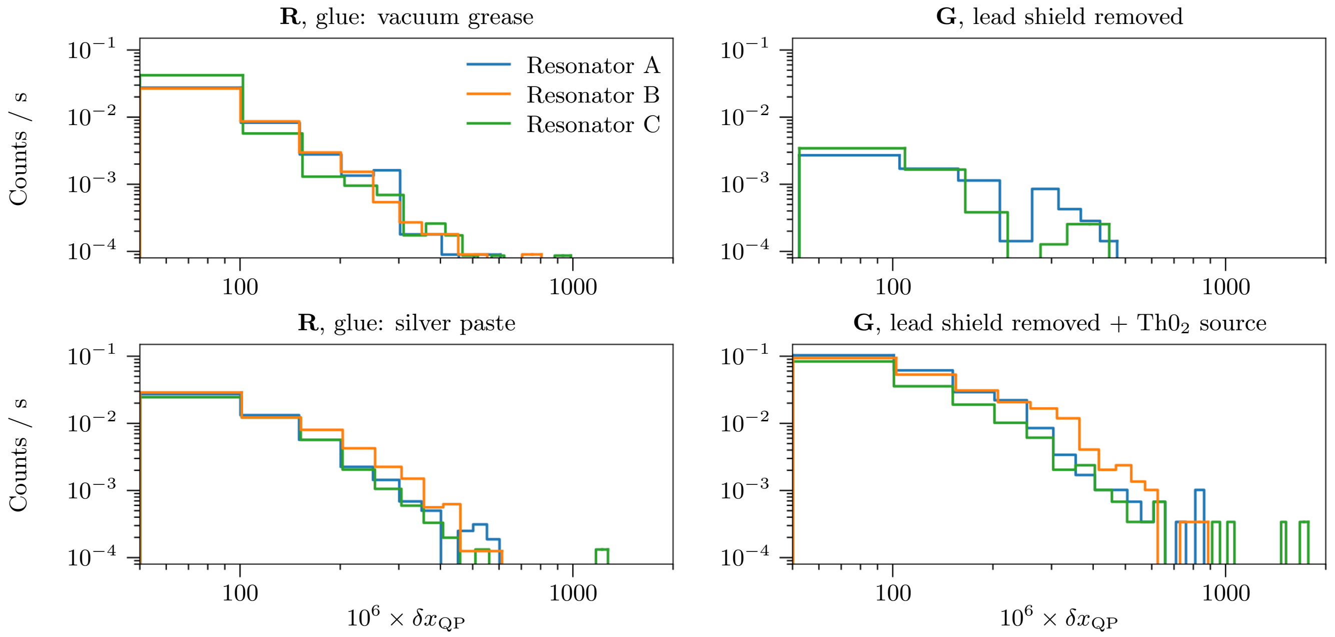

Supplementary Figure 7. **Distribution of QP bursts in R and G**. The bin size is 5×10^{-5} in all panels. For the G setup with the Pb shield removed, data were not available for resonator B due to technical and time constraints.

Supplementary Note 8: Phonon absorption efficiency

The Cooper pair condensate of a resonator absorbs only a small fraction ϵ of the total energy deposited in the substrate chip. This fraction depends, among others, on the material and volume of the superconductor, its interface with the substrate, the material and geometry of the substrate and its coupling to the thermal bath. As a consequence, the efficiency of energy absorption cannot be calculated a priori but requires a dedicated measurement.

To our knowledge, there are no published results on the absorption efficiency of a grAl film deposited on a sapphire substrate. Due to the phonon acoustic mismatch between grAl and the sapphire substrate, we expect the efficiency ϵ to be very small. To constrain this parameter without contaminating the prototype, we exposed the chip to a removable ThO_2 source and compared the calculated spectrum of the energy deposited in the substrate with the measured spectrum of the energy absorbed by the three resonators.

In Supplementary Figure 8a we show the distribution of energy absorbed by the resonators in G with the Pb shield removed, and exposed to a ThO_2 source. We performed a GEANT4 [8–11] based simulation to estimate the energy released in the substrate by the source. The simulation includes the sapphire chip, its copper holder, and the cryostat shields. The activity of the source was not precisely known, so we did not normalize the simulated events by time. We plot the results of the simulation in Supplementary Figure 8b. We can estimate the efficiency ϵ by dividing the average energy deposited in the resonator by the average energy deposited in the substrate. We obtain $\epsilon \sim 10^{-4}$, 10^{-3}, and 0.5×10^{-4} for resonator A, B, and C, respectively.

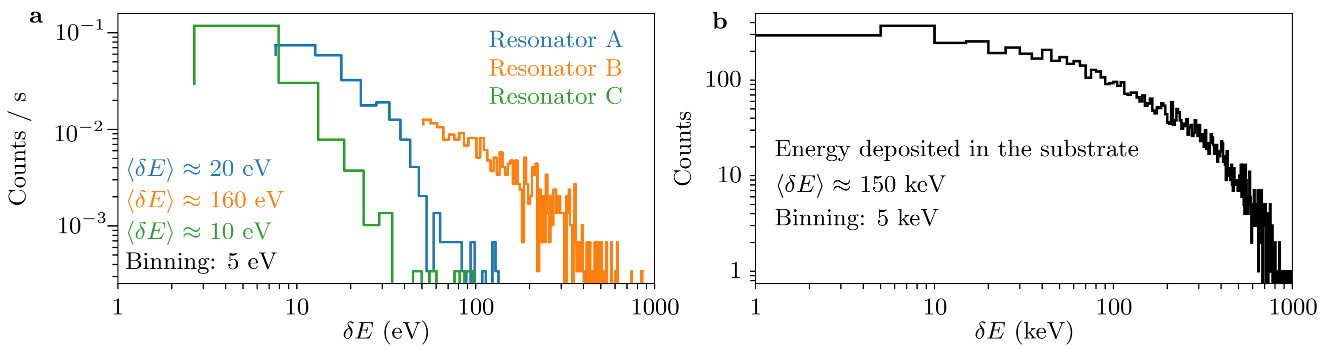

Supplementary Figure 8. **Simulation of resonator response under ThO_2 exposure.** **a**, distribution of the energy absorbed in the resonators in G under ThO_2 exposure. **b**, Monte Carlo simulation of the energy deposited by a ThO_2 source in the sapphire substrate.

This value could appear very small compared to values reported in Ref. [12–14], therefore, to asses the phonon impedance mismatch at the substrate-film interface we performed a separate experiment in R. We measured a sample consisting of a 60 nm thick grAl kinetic inductance detector (KID) deposited on on a 2×2 cm^2, 330 µm thick sapphire substrate. The design was similar to the one described and depicted in Ref. [14], with an active surface of 2 mm^2. The chip was assembled in a copper holder hosting a ^{55}Fe X-ray source, emitting Mn K-alpha characteristic X-rays at 5.9 and 6.4 keV with a rate of ~100 mHz. Due to their low energy, the X-rays are completely absorbed in the substrate. This type of source has the disadvantage that, being permanently exposed to the sample, prevents the achievement of low-radioactivity levels. On the other hand, it produces events of well defined energy, in contrast to the broad spectrum emitted by the removable ThO_2 source (Supplementary Figure 8b). This allows to obtain a precise measurement of the efficiency. Following the methods outlined in Refs. [4, 14] we can write the grAl KID efficiency ϵ as:

$$\epsilon = \frac{N_0 V \Delta_0^2}{\alpha S_2(\omega, T) Q} \frac{\delta\phi}{E_{\mathrm{nom}}} \tag{3}$$

where:

- $N_0 = 1.72 \times 10^{10}$ eV^{-1}µm^{-3} is the density of states at the Fermi level, assumed to be the same as that of aluminum;
- V is the supercurrent mode volume of the resonator (a 2×2 mm^2 wide and 60 nm thick KID);
- $\Delta_0 = 1.55\times10^{-4}$ eV is the superconducting gap of the grAl film employed for this measurement (note that $\Delta_0 = 1.65\times10^{-4}$ eV for resonators A, B, and C);

- $\alpha = 0.4$ is the kinetic inductance fraction of the grAl resonator, evaluated using the Sonnet finite element simulator [15];
- Q is the loaded quality factor of the resonator, extracted using a fit to the resonant circle [16];
- $S_2(\omega,\mathrm{T})$ is a slow function of the resonant frequency and of the effective temperature, derived from the Mattis-Bardeen theory [17]. Its value usually ranges from 2 to 4, and for the grAl KID resulted 2.27;
- $\delta\phi = 18.2$ mrads is the phase variation corresponding to the energy deposited by the ^{55}Fe source (E_{nom}), extracted from a fit to the ^{55}Fe peak.

Substituting these values into the previous formula we obtain the grAl KID efficiency of 0.32%.

We would like to emphasize that this value cannot be easily scaled to the A-C resonators discussed in the main text, which have 2-3 orders of magnitude smaller supercurrent mode volume. Even if the efficiency scales almost linearly with the mode volume of the KID [13], an extrapolation to a mode volume smaller by 2-3 orders of magnitude would not be reliable. Thus, we interpret the calibrated grAl KID efficiency of 3.2×10^{-3} as an upper bound to the efficiency of the A-C resonators.

Supplementary Note 9: Background Sources

In this section we budget the counting rate due to cosmic rays and environmental radioactivity in the laboratories where the measurements were performed. We used the GEANT4 toolkit to simulate interactions in the sapphire chip and its copper enclosure. The cryostats and the magnetic shield were schematically modelled as a series of nested vessels made of copper, aluminum, and μ-metal.

The absolute muon flux in the R and K laboratories is expected to be ∼1 μ/cm^2/min. We simulated 10^9 muons with energy and angular distribution reported in Ref. [18]. The resulting rate of energy deposits in the sapphire chip at R is 0.6 mHz, with an average energy of 0.8 MeV. This value includes the interactions of both muons and secondary particles produced by muons interacting in the material close to the device. In K we predict a ∼20% lower flux because, in contrast to R, the cryogenic facility is located in the basement and thus is partly shielded by the building above and the ground surrounding it. The G facility benefits from a 1.4 km thick rock overburden, which suppresses the flux of cosmic rays by six orders of magnitude. We highlight that in all the experimental facilities, the interaction rate due to cosmic rays is negligible compared to the measured one.

A major source of QP bursts is environmental radioactivity. Its contribution was measured through a cylindrical NaI γ-spectrometer (3″ diameter and height) in all the measurements sites. In G we also repeated the assay by surrounding the spectrometer with the same 10 cm lead shield used during the measurements with resonators. The results are shown in Supplementary Figure 9.

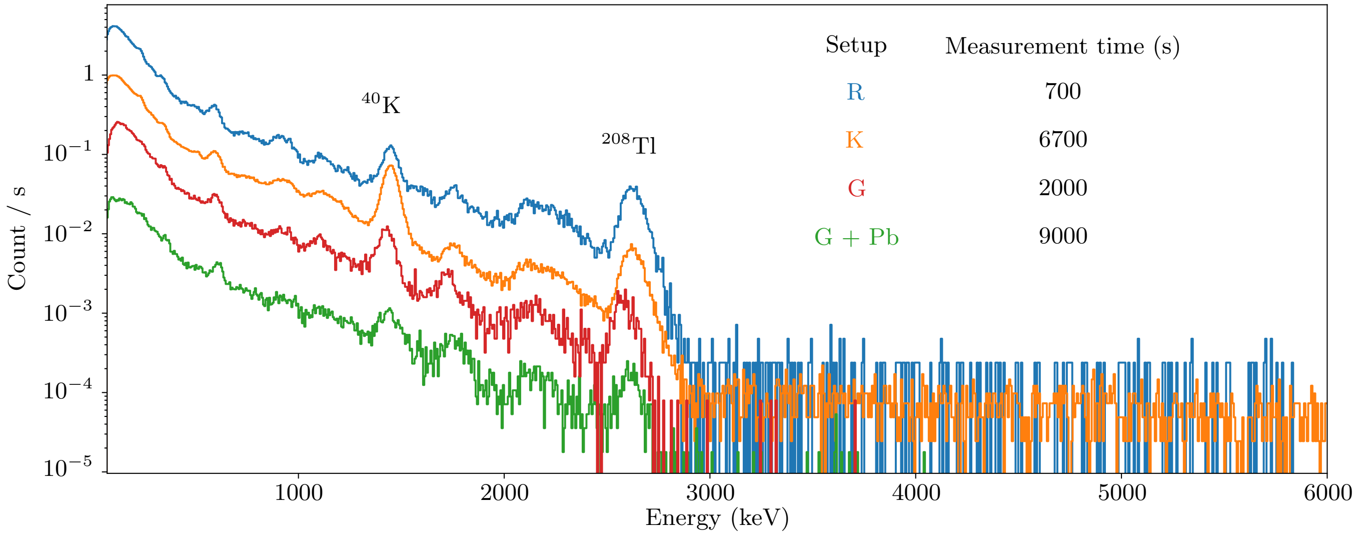

Supplementary Figure 9. **Measurement of environmental radioactivity.** Histograms of NaI scintillation spectroscopy performed in the R (blue), K (orange), and G (red) setups. In G we repeated the measurement surrounding the NaI detector with the same lead shield used for the measurements (green), suppressing the laboratory radioactivity by almost a factor 8. All the spectra show the signatures of ^{40}K (1.46 MeV peak), of isotopes belonging to the Thorium chain (e.g., the 2.6 MeV peak of ^{208}Tl) and to the Uranium chain (e.g., the 1.76 MeV peak of ^{214}Bi).

In each spectrum, we could clearly identify the typical signatures of ^{40}K, ^{232}Th, ^{238}U and their daughters, the most common ambient contaminants. We constructed a background model of the G setup by generating ^{40}K, ^{232}Th and ^{238}U uniformly distributed on a spherical surface around the NaI spectrometer, and by simulating the interactions of their decay products in the detector. The relative weights and rates of the contaminants were fixed by fitting the results of the simulation to the measured spectrum. We obtained an absolute γ flux in the G site of 0.6 γ cm^{-2} s^{-1} This flux was used as input for the simulation to predict the rate of energy deposits in the chip. We set the same threshold in the simulated spectrum and in the spectrum measured with the three resonators (i.e. 60 keV). However, we repeated the same analysis varying the energy threshold up to 120 keV and obtained consistent results. Our simulation predicts that, in G, the laboratory radioactivity is responsible for a rate of energy deposits of 4 ± 1 mHz; such rate has to be compared with the average rate of QP bursts measured by the resonators of 6.6 ± 0.7 mHz. We followed a similar procedure to predict the rates due to laboratory contamination in the other measurement sites. The expected laboratory contribution to the chip rate after shielding the cryostat with the 10 cm thick lead shield is 0.5

$\pm$ 0.1 mHz. Comparing this value to the measured one, 2.6 $\pm$ 0.6 mHz, we observe a mild excess in the experimental rate, probably related to residual contaminations of the prototype materials. The laboratory contribution to the chip rate in R was estimated to be 48 $\pm$ 11 mHz, while the measured rate resulted 44 $\pm$ 5 mHz (vacuum grease) and 52 $\pm$ 4 mHz (silver paste). Finally, we observe that the chip rate ascribed to the laboratory radioactivity in K was 16 $\pm$ 4 mHz, largely below the measured value of 76 $\pm$ 1 mHz. Such excess is likely ascribed to the lack of protocols for the suppression of materials contamination that, as explained in the main text, were adopted only after the measurements in K. As so, future qubits experiments aiming at low radioactivity levels could have to cope with the radio-assay of all the materials used for the device operation.

[1] Pop, I. M. *et al.* Fabrication of stable and reproducible submicron tunnel junctions. *Journal of Vacuum Science & Technology B* **30**, 010607 (2012).
[2] Stehli, A. *et al.* Coherent superconducting qubits from a subtractive junction fabrication process. *Applied Physics Letters* **117**, 124005 (2020).
[3] Swenson, L. J. *et al.* High-speed phonon imaging using frequency-multiplexed kinetic inductance detectors. *Applied Physics Letters* **96**, 263511 (2010).
[4] Moore, D. C. *et al.* Position and energy-resolved particle detection using phonon-mediated microwave kinetic inductance detectors. *Applied Physics Letters* **100**, 232601 (2012).
[5] Bourrion, O. *et al.* NIKEL: Electronics and data acquisition for kilopixels kinetic inductance camera. *Journal of Instrumentation* **7**, P07014–P07014 (2012).
[6] Hickish, J. *et al.* A decade of developing radio-astronomy instrumentation using casper open-source technology. *Journal of Astronomical Instrumentation* **05**, 1641001 (2016).
[7] Shahid, S. *et al.* Reflection type Q-factor measurement using standard least squares methods. *IET Microwaves, Antennas & Propagation* **5**, 426 (2011).
[8] GEANT-4: a toolkit for the simulation of the passage of particles through matter. https://geant4.web.cern.ch.
[9] Agostinelli, S. *et al.* Geant4—a simulation toolkit. *Nucl. Instrum. Methods Phys. Res. A* **506**, 250 – 303 (2003).
[10] Allison, J. *et al.* Geant4 developments and applications. *IEEE Transactions on Nuclear Science* **53**, 270–278 (2006).
[11] Allison, J. *et al.* Recent developments in geant4. *Nucl. Instrum. Methods Phys. Res. A* **835**, 186 – 225 (2016).
[12] Moore, D. C. A search for low-mass dark matter with the cryogenic dark matter search and the development of highly multiplexed phonon- mediated particle detectors. *Ph.D. thesis, California Institute of Technology, Pasadena* (2012).
[13] Cardani, L. *et al.* New application of superconductors: High sensitivity cryogenic light detectors. *Nuclear Instruments and Methods in Physics* **845**, 338–341 (2016).
[14] Cardani, L. *et al.* High sensitivity phonon-mediated kinetic inductance detector with combined amplitude and phase read-out. *Applied Physics Letters* **110**, 033504 (2017).
[15] Sonnet Software. https://www.sonnetsoftware.com.
[16] Casali *et al.* Characterization of the kid-based light detectors of calder. *Journal of Low Temperature Physics* **184**, 142–147 (2016).
[17] Gao, J. *et al.* Equivalence of the effects on the complex conductivity of superconductor due to temperature change and external pair breaking. *Journal of Low Temperature Physics* **151**, 557–563 (2008).
[18] Shukla, P. & Sankrith, S. Energy and angular distributions of atmospheric muons at the earth. *arXiv:1606.06907* (2018).
